# Improving the Outcome of Patients With Heart Failure: Assessment of Iron Deficiency and Intravenous Iron Replacement

**DOI:** 10.7759/cureus.47027

**Published:** 2023-10-14

**Authors:** Hassan O Yera, Ahsan Khan, Olawale M Akinlade, Asgher Champsi, Van Nam J Glouzon, Charles Spencer

**Affiliations:** 1 Internal Medicine, The Shrewsbury and Telford Hospital NHS Trust, Telford, GBR; 2 Cardiology, Heart and Lung Centre, New Cross Hospital, Wolverhampton, GBR; 3 Cardiology, Dumfries and Galloway Royal Infirmary, Dumfries, GBR; 4 Internal Medicine, Heart and Lung Centre, New Cross Hospital, Wolverhampton, GBR

**Keywords:** morbidity and mortality, heart failure with reduced ejection fraction, quality improvement, ferric carboxymaltose, iron deficiency anaemia

## Abstract

Background

Iron deficiency (ID) has been shown to be a significant co-morbidity in patients with heart failure (HF), independent of their anaemia status. Correction of ID has been shown to improve quality of life, recurrent heart failure hospitalizations and morbidity. A quality improvement project was designed to improve the assessment and treatment of iron deficiency in HF patients in our tertiary care centre.

Methods and results

An initial baseline dataset was collected, followed by two cycles of interventions to help improve the care of HF patients admitted to our hospital over a two-month period. The Plan-Do-Study-Act (PDSA) cycle approach was applied, with the first intervention involving raising awareness of the importance and need to assess the iron status of HF patients through education provided to doctors, nurses and patients. Furthermore, information leaflets were produced and disseminated across the medical wards and through social media forums. The post-intervention datasets were collected and compared to the baseline outcomes. Baseline data showed that only four (20%) of heart failure patients had their iron status checked. Following the interventions, screening for ID increased to 80% (16), of which 85% (11) of those who identified as iron deficient received intravenous iron replacement.

Conclusion

The project was successful in improving the practice of screening for iron deficiency and intravenous replacement of iron in patients with HF.

## Introduction

Problem

Patients with heart failure (HF) often suffer from acute decompensation requiring hospitalisation [1,2]. Recurrent hospitalisation has been associated with increased mortality and reduced quality of life. Several co-morbidities have been associated with recurrent decompensation and hospitalisation including iron deficiency [1,2]. Intravenous iron replacement has been associated with reduced hospitalisation and improved quality of life [1,2].

New Cross Hospital is a tertiary centre in the West Midlands [3]. It has a state-of-the-art heart and lung centre that admits patients with HF either straight into the cardiology ward or to the acute medical unit. Patients with HF are also admitted directly from the clinic or other medical wards following review by the in-reach heart failure team.

We noticed that most patients who were admitted with HF, particularly those with reduced ejection fraction. do not have their iron status assessed using serum ferritin and transferrin saturation. Additionally, those patients found to be iron deficient following investigation for low haemoglobin received iron supplementation via oral route rather than intravenous route using ferric carboxymaltose, which is less efficacious [4].

We therefore undertook a quality improvement project (QIP) aiming to improve the assessment and appropriate correction of iron deficiency in this population of patients.

Background

Iron deficiency, which can be present regardless of anaemia, is present in up to 55% of chronic HF (CHF) patients and in up to 80% of those with acute HF (AHF) [1,2,5,6]. This has been shown to be associated with reduced exercise capacity, recurrent HF hospitalisations and high cardiovascular (CV) and all-cause mortality [7,8]. Although the exact cause of iron deficiency in HF is not clear yet, possible mechanisms include increased loss, reduced intake, reduced absorption due to malnutrition and decreased gut absorption and impaired metabolism caused by the chronic inflammatory activation of HF. An important factor is the effect of hepcidin, a chemical released by the liver in chronic inflammatory states. Hepcidin acts on the gut and the reticuloendothelial system to prevent the absorption of iron and release of stored iron, respectively, making the oral route a less favourable option for the correction of iron deficiency [7-9]. The deficiency state thus created may impair functional capacity, precipitate circulatory decompensation, promote skeletal muscle dysfunction and is associated with frailty regardless of the presence of anaemia.

The World Health Organisation (WHO) defines anaemia as a haemoglobin concentration of <12 g/dL in women and <13 g/dL in men. Iron deficiency in patients with HF is defined as either serum ferritin concentration of <100 ng/mL or 100-299 ng/mL with transferrin saturation (TSAT) <20% [9-11].

Numerous randomised control trials (RCTs) have shown that iron supplementation with intravenous ferric carboxymaltose is safe and improves symptoms, exercise capacity and quality of life of patients with HF and iron deficiency [11-14]. The effects of supplementation have been shown to be favourable, with minimal side effects [15]. Meta-analyses of RCTs have also shown that correction of iron deficiency led to a reduction in the risk of all-cause death or CV hospitalization, CV death or HF hospitalisation, or CV death or recurrent CV or HF hospitalisations [16,17].

The European Society of Cardiology, in its 2021 guidelines for the diagnosis and treatment of acute and chronic heart failure, therefore recommended that heart failure patients be periodically screened for iron deficiency and anaemia with full blood count, serum ferritin and TSAT and that intravenous iron supplementation with ferric carboxymaltose should be considered in symptomatic patients with left ventricular ejection fraction (LVEF) < 50% and iron deficiency, defined as serum ferritin <100 ng/mL or serum ferritin 100-299 ng/mL with TSAT <20%, to alleviate HF symptoms and improve exercise capacity and quality of life (QOL) [18].

This article was previously presented as a clinical abstract presentation at the British Cardiovascular Society Centenary Conference on June 6, 2022.

## Materials and methods

Baseline measurements

A baseline data collection was done retrospectively, looking at patients who were recently discharged following inpatient management for acute decompensated HF with reduced ejection fraction. A total of 20 patients had the following data collected: date of admission, date of discharge, recent echo ejection fraction, HF medications, if iron status was checked using serum ferritin and TSAT, if iron was supplemented and the route used for supplementation. This information was obtained from scanned patient notes, recent discharge letters, recent investigations and electronic prescribing software.

The data showed that 80% (16) of patients were not screened for iron deficiency. Three of the patients who had their iron status checked had iron deficiency, with two of them going on to have iron supplementation via the intravenous route.

Design

The outcome of the baseline data clearly showed the need for an improvement in the assessment of iron deficiency in patients admitted with HF. We, therefore, set out to improve by 80% the screening of patients with HF for iron deficiency and the utilisation of intravenous ferric carboxymaltose to replete those found to be iron-depleted over an eight-week period.

A total of 20 patients were included in each cycle. Our outcome measure was the percentage of patients who had iron studies done and those who had intravenous iron replacement. The data in each cycle was collected retrospectively from the electronic prescribing system, the scanned patient notes and the discharge letters. Only heart failure patients with ejection fraction <50% as defined by the 2021 European Society of Cardiology guideline for the management of acute and chronic heart failure were included in this project [18].

Our strategies focused on addressing this issue by educating doctors and advanced nurse practitioners via face-to-face teaching on current trends and recent guidelines on the management of HF with an emphasis on iron deficiency as a co-morbidity. This intervention was reinforced with the creation of posters that were disseminated to medical wards and via WhatsApp.

The Plan-Do-Study-Act (PDSA) quality improvement model was used for this project. After the first PDSA cycle, a week was given for the intervention to integrate into the daily practice of the doctors. Data were then collected retrospectively for two weeks, and the results were analysed. PDSA cycle two began a week later and a similar process was repeated.

The project was led by the primary author and supported by co-authors. Patients were identified on discharge and details required for analysis were collected anonymously using a pre-designed proforma. Details collected include diagnosis, ejection fraction, iron status assessment and if intravenous iron was infused. To ensure anonymity and avoid duplicates, age and sex were used to differentiate patients. If these two parameters matched other data, we looked back into records to ensure the same patient was not admitted twice during the project period.

The HF nurses were involved in the intervention and played a significant role in educating doctors in other medical wards, thus ensuring patients had iron supplementation before discharge.

Lastly, we involved the acute medical department through which most of the patients are usually admitted. They were instrumental in ensuring that patients who were discharged home before admission to the cardiology ward were screened for iron deficiency and received intravenous iron replacement if iron deficient.

Strategy

Our SMART (specific, measurable, achievable, realistic and time-bound) aim was to improve the screening of HF patients for iron deficiency and repleting those found to be deficient with intravenous iron regardless of anaemia status by 80%. We undertook two PDSA test cycles.

PDSA Cycle One

Our initial intervention focused on educating the junior doctors and advanced nurse practitioners in the cardiology ward and the acute medical unit on the need for the regular assessment of iron status in patients admitted with HF. The importance of iron supplementation in HF patients who were found to be deficient was emphasised vis-à-vis the reduction in hospitalisation, the improvement of symptoms and QOL.

PDSA Cycle Two

**Figure 1:**
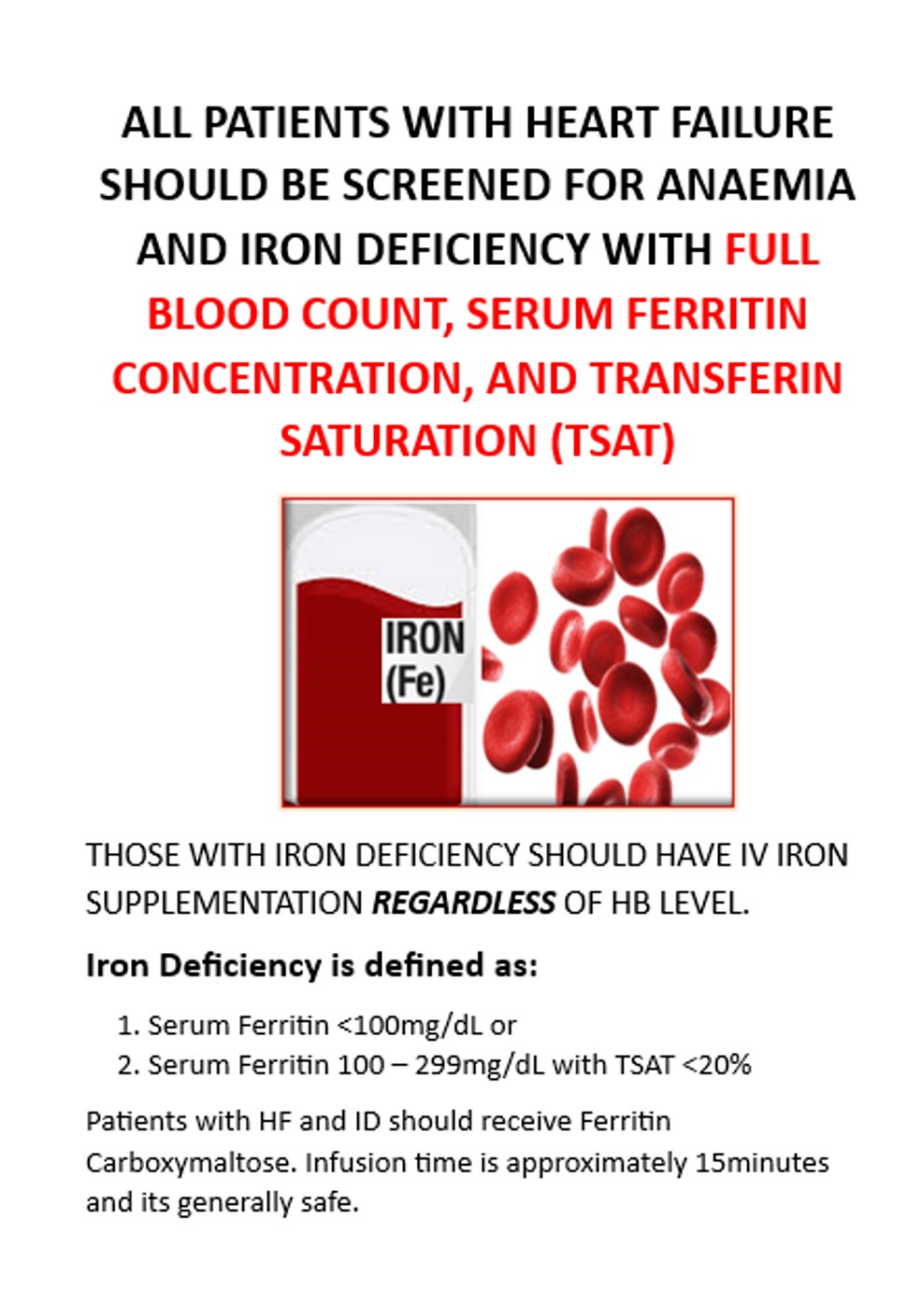
Poster reminding doctors and ANPs to assess patients for ID and treat ID with ferrous carboxymaltose ANP, Advanced Nurse Practitioners; ID, Iron Deficiency; HB, Haemoglobin; HF; Heart Failure

To build on the strength of cycle one achievement, we created wall posters to serve as aid memoirs for doctors and advanced nurse practitioners. The posters summarised the pathway to diagnosis of iron deficiency in HF patients and the criteria for diagnosis (Figure 1). These posters were disseminated to the wards and via WhatsApp. In addition, the HF team were given posters to put in their outreach file when going to review patients to serve as a continuous reminder for them to ensure that HF patients they reviewed on medical wards are assessed and, where necessary, prescribed intravenous iron regardless of anaemia status. They also provided continuous reminders to doctors in various medical wards.

## Results

The interventions had significant effects on the outcome that we set out to measure. Our outcome measures were to increase the number of patients who had their iron status assessed and to increase the number of patients who received intravenous iron.

**Figure 2:**
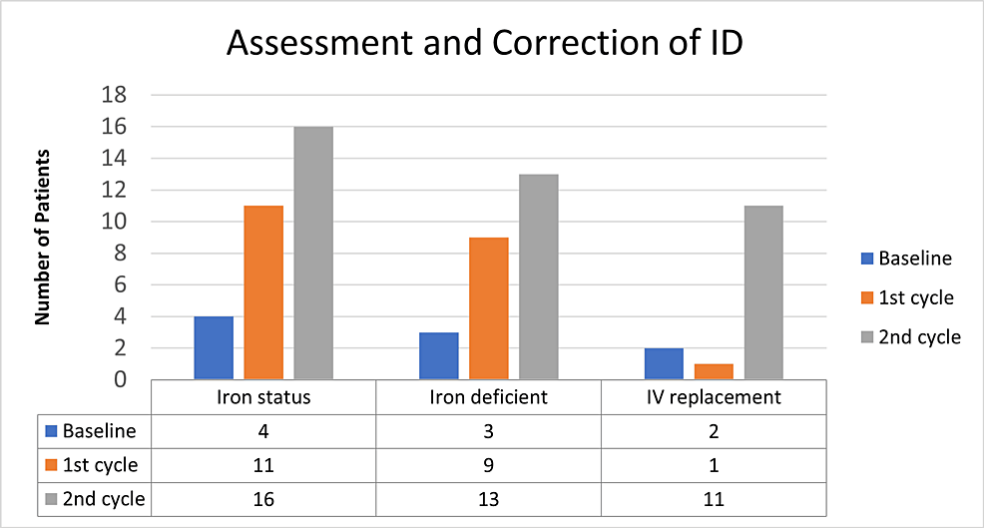
Impact of interventions on the assessment and correction of iron deficiency intravenously IV, Intravenous; ID, Iron Deficiency

At baseline, the proportion of heart failure patients who had their iron status assessed was 20% (4). Following interventions, this increased to 55% (11) in cycle one and to 80% (16) in cycle two. The proportion of those found to be iron-deficient increased as the proportion of those screened for iron deficiency increased. From only three patients of the 20 screened at baseline, to 13 after cycle two. Overall, a significant proportion of those screened during the project had iron deficiency as shown in Figure 2.

The proportion of those who had intravenous iron replacement increased significantly from baseline after interventions (Figure 2). At baseline, two of the three found to be iron deficient had intravenous iron replacement. After initial intervention, there was no significant improvement, as only one of the nine found to be iron deficient after cycle one had IV iron replacement. However, after cycle two, 85% (11) of the 13 found to have ID had IV iron replacement.

## Discussion

This quality improvement project aimed at improving by 80%; the routine assessment of iron status and intravenous replacement of iron in heart failure patients with iron deficiency led to significant improvements in practice. At the end of our second PDSA cycle, most patients admitted with heart failure were screened for iron deficiency and a significant number of those found to be deficient had intravenous iron replacement. Adherence with this guideline-recommended treatment of heart failure patients with reduced ejection fraction will potentially lead to an improvement in quality of life and a reduction in hospitalisation and morbidity [13,16,18].

Our baseline analysis showed that patients were being discharged without an assessment of their iron status or supplementation with iron transfusion if found deficient, despite existing guidelines recommending otherwise. Data from the Swedish Heart Failure Registry reported less than 25% of HF patients were screened for iron deficiency before discharge [19]. This suggests efforts are needed to ensure the translation of guidelines to daily practice.

Our project has shown that through the implementation of several interventions using the Plan-Do-Study-Act model, the quality of care delivered to patients can be improved. Our main interventions, which involved education and reminders using wall posters and WhatsApp, were the key drivers for change. These led to improved practice, as the majority of the HF patients admitted had iron status assessed and most with ID had intravenous iron replacement. Educating doctors and other healthcare practitioners about the higher cut-off for iron deficiency in heart failure was paramount, as patients with HF are considered to have anaemia of chronic disease in addition to having a high circulating hepcidin produced by the liver in this chronic inflammatory state. In addition, emphasis was placed on the poor absorption of oral iron due to gut oedema [4,15].

The PDSA approach to quality improvement allowed us to test interventions that will fit our setting such as the use of social media, arranging education sessions and dissemination of knowledge and guidelines directed care via posters, and importantly, the involvement of stakeholders. Significant stakeholders identified in this project include heart failure nurses, advanced nurse practitioners and ward pharmacists. The heart failure nurses were instrumental in disseminating and encouraging the practice in the cardiology ward and other medical wards thus further ensuring a change in practice.

An Important limitation we encountered while carrying out this project was the rotations of junior doctors. As this could potentially affect the positive change already made, we ensured posters were placed in visible places to educate and remind them.

To maintain the change and to address the limitations, the outcome of the project was presented to the local cardiology governance meeting where a decision was made to set up a designated area for iron infusion for patients that are routinely screened in the outpatient clinic and those patients who are fit for discharge to avoid unnecessary bed occupancy. In addition, the local trust guideline was to undergo revision to include screening of HF patients for iron deficiency and the creation of a laboratory HF request bundle that will include ferritin and transferrin saturation.

Furthermore, the HF team will carry out re-audits to ensure continuous improvement in the assessment of iron status and appropriate replacement. The cardiac advanced nurse practitioners who are substantive employees of the trust will provide regular reminders to doctors via WhatsApp. This will be in addition to the wall posters that have been strategically placed in the wards.

## Conclusions

This project has demonstrated improvement in the management of iron deficiency in HF patients. We were able to achieve our SMART aim using two PDSA cycles, with our main interventions being education and poster reminders via face-to-face meetings and WhatsApp. To sustain this, the outcome was presented in the cardiology governance meeting where processes that will continue to ensure sustainability were planned, which included creating a designated area for intravenous iron infusion, updating local guidelines and regularly auditing the care of HF patients by the HF team.

We have demonstrated that our approach has improved the care of patients with heart failure, and we believe that if they are replicated in other settings, they will yield positive results.
